# 2430. Risk Factors for Mortality among Patients who Present from the Community with Central Line-Associated Bloodstream Infections

**DOI:** 10.1093/ofid/ofad500.2049

**Published:** 2023-11-27

**Authors:** Opeyemi Oladapo-Shittu, Patrick R Ching, Yea-Jen Hsu, Heather Saunders, Alejandra B Salinas, Taylor N Helsel, Stephanie Mayoryk, Avinash Gadala, Sara E Cosgrove, Clare Rock, Eili Klein, Lisa Maragakis, Lisa Pineles, Anthony Harris, Carlos Mejia-Chew, Sara C Keller

**Affiliations:** Johns Hopkins University, Baltimore, Maryland; Washington University School of Medicine, Saint Louis, Missouri; Johns Hopkins Bloomberg School of Public Health, Baltimore, Maryland; Johns Hopkins University, Baltimore, Maryland; Johns Hopkins University School of Medicine, Baltimore, Maryland; Johns Hopkins University School of Medicine, Baltimore, Maryland; University of Maryland School of Medicine, Shrewsbury, Pennsylvania; Johns Hopkins Health System, Baltimore, Maryland; Johns Hopkins School of Medicine, Baltimore, MD; Johns Hopkins School of Medicine, Baltimore, MD; Johns Hopkins School of Medicine, Baltimore, MD; Johns Hopkins Medicine, Baltimore, MD; University of Maryland School of Medicine, Shrewsbury, Pennsylvania; University of Maryland School of Medicine, Shrewsbury, Pennsylvania; Washington University in St Louis, St. Louis, Missouri; Johns Hopkins University School of Medicine, Baltimore, Maryland

## Abstract

**Background:**

Central line-associated bloodstream infections (CLABSI) lead to significant morbidity and mortality. Surveillance has contributed to reductions in the CLABSI rate in acute care hospitals. Although there are increasing numbers of patients with central venous catheters outside of acute care hospitals, the prevalence of and mortality risk associated with CLABSIs present-on-admission (POA) to acute care hospitals is unknown.

To explore the burden of CLABSI POA and the risk factors associated with an all-cause mortality.

**Methods:**

We identified patients presenting to hospitals with CLABSI (regardless of pre-admission location) in two large health systems based in Maryland, Washington DC, and Missouri between November 2020-October 2021. CLABSI was defined using an adaptation of the acute care CLABSI definition.

**Results:**

Out of 402 patients identified with CLABSI POA, in-hospital mortality was 10.4%. Within six months of hospital presentation, 126 patients died (31.3%) (Figure 1). The median Pitt bacteremia score was 1 (interquartile range: 0-2), with 82 (20.4%) admitted to the adult intensive care unit (ICU), and 9 (2.2%) admitted to the pediatric ICU. The mean hospital length of stay was 8 days (interquartile range: 5-14). Compared with patients < 45 years of age, patients aged 45-64 and >65 had increased mortality within 6 months (OR: 3.49, 95% CI: 1.53-7.96; OR: 5.76, 95% CI: 2.24-14.81, respectively). Compared with patients receiving Medicaid, patients who were self-pay had increased mortality within 6 months (OR: 4.18, 95% CI: 1.13-15.43). Receipt of chemotherapy within the last 6 months and hospital discharge ≤ 30 days before presentation were associated with increased mortality within 6 months (OR: 2.53, 95% CI: 1.12-5.71; OR: 1.19, 95% CI: 1.13-3.23; respectively) (Table 1).

**Figure:**
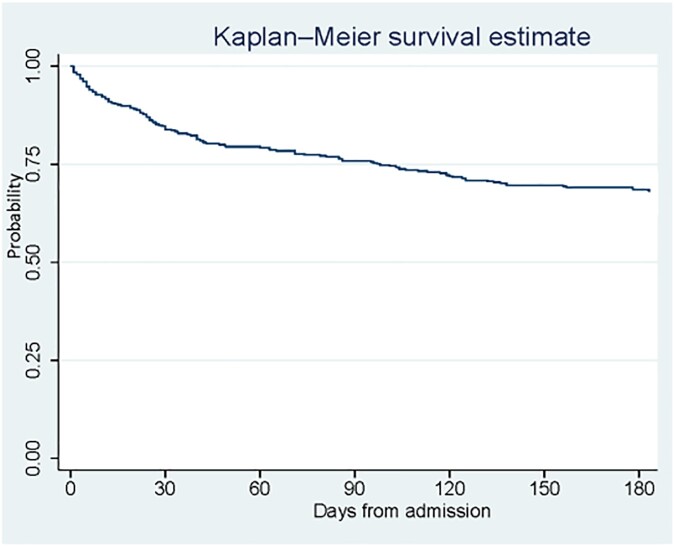

**Figure:**
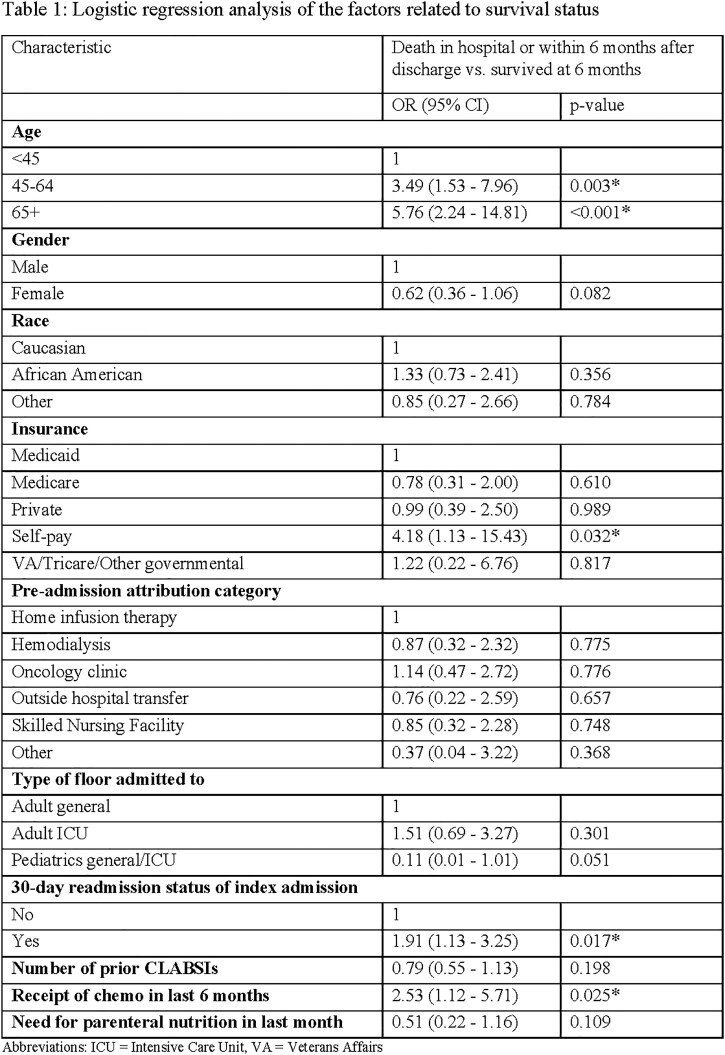

**Conclusion:**

CLABSI POA was associated with a high mortality, with almost one in three patients deceased within 6 months. Patients at risk of higher mortality included those over the age of 44, self-pay insurance, receipt of chemotherapy in the last 6 months, and recent hospital discharge. These results underscore the need for CLABSI prevention initiatives outside acute care settings.

**Disclosures:**

**Stephanie Mayoryk, MAS BSN RN CIC**, PDI: Honoraria **Sara E. Cosgrove, MD, MS**, Debiopharm: Advisor/Consultant|Duke Clinical Research Institute: Advisor/Consultant **Carlos Mejia-Chew, MD**, INSMED: Grant/Research Support|RevImmune: Grant/Research Support **Sara C. Keller, MD, MPH, MSPH**, Pfizer: Advisor/Consultant

